# Influence of crystal structure of nanosized ZrO_2_ on photocatalytic degradation of methyl orange

**DOI:** 10.1186/s11671-015-0780-z

**Published:** 2015-02-18

**Authors:** Sulaiman N Basahel, Tarek T Ali, Mohamed Mokhtar, Katabathini Narasimharao

**Affiliations:** Department of Chemistry, Faculty of Science, King Abdulaziz University, P. O. Box, 80203, Jeddah, 21589 Kingdom of Saudi Arabia; Chemistry Department, Faculty of Science, Sohag University, P.O. Box 82524, Sohag, 82524 Egypt; Physical Chemistry Department, National Research Centre, El Buhouth St., Dokki, Cairo, 12622 Egypt

**Keywords:** ZrO_2_, Structural effect, Photocatalytic degradationl, Methyl orange

## Abstract

Nanosized ZrO_2_ powders with near pure monoclinic, tetragonal, and cubic structures synthesized by various methods were used as catalysts for photocatalytic degradation of methyl orange. The structural and textural properties of the samples were analyzed by X-ray diffraction, Raman spectroscopy, TEM, UV-vis, X-ray photoelectron spectroscopy (XPS), and N_2_ adsorption measurements. The performance of synthesized ZrO_2_ nanoparticles in the photocatalytic degradation of methyl orange under UV light irradiation was evaluated. The photocatalytic activity of the pure monoclinic ZrO_2_ sample is higher than that of the tetragonal and cubic ZrO_2_ samples under optimum identical conditions. The characterization results revealed that monoclinic ZrO_2_ nanoparticles possessed high crystallinity and mesopores with diameter of 100 Å. The higher activity of the monoclinic ZrO_2_ sample for the photocatalytic degradation of methyl orange can be attributed to the combining effects of factors including the presence of small amount of oxygen-deficient zirconium oxide phase, high crystallinity, large pores, and high density of surface hydroxyl groups.

## Background

The rapid growth of the textile industry has led to the accumulation of various organic pollutants, with dyes accumulating in bodies of water as a particularly severe example. This type of aquatic pollution has indirect or direct adverse effects on the biosphere [1]. Photocatalysis is one promising approach to protect the aquatic environment based on its ability to oxidize low concentrations of organic pollutants in water [2,3]. In the past two decades, many oxide and sulfide semiconductors such as TiO_2_, ZnO, WO_3_, SrTiO_3_, ZnS, and CdS were applied as photocatalysts for environmental control technology and also a wide range of chemical reactions [4]. Recently, Kuriakose et al. [5,6], Cheng et al. [7], and Ren et al. [8] successfully employed ZnO- and TiO_2_-based nanomaterials for photocatalytic degradation of organic dyes. ZrO_2_ has been considered as a photocatalyst in different chemical reactions due to its relatively wide band gap value *E*_g_ and the high negative value of the conduction band potential [9]. The reported band gap energy of ZrO_2_ range was between 3.25 and 5.1 eV, depending on the preparation technique of the sample [10].

It is reported that a good manipulation of ZrO_2_ morphological tuning, porous structure control, and crystallinity development is required in order to enhance the light harvesting capability, prolong the lifetime of photoinduced electron-hole pairs, and facilitate the reactant accessibility to surface active sites [11]. As ZrO_2_ is used in a wide variety of applications in addition to photocatalysis, the fabrication of identical ZrO_2_ nanoscale structures has been recently attracted a great deal of interest. Nanocrystalline ZrO_2_ with various attractive morphologies has been effectively prepared by different synthesis methods like hydrothermal synthesis, sol-gel synthesis, precipitation, and thermal decomposition [12].

It is well known that ZrO_2_ has three polymorphs [13]: monoclinic, tetragonal, and cubic. Preparation methods play an important role in determining the final crystal structure of ZrO_2_. Although the different surface properties on different ZrO_2_ polymorphs have been extensively studied [14,15], the effect of crystal structures on photocatalysis has rarely been investigated.

Nawale et al. [16] synthesized ZrO_2_ samples using thermal plasma reactor at different operating pressures. The sample which contained both tetragonal and monoclinic phases synthesized at 1.33 bar of operating pressure showed the highest photocatalytic activity. The presence of tetragonal phase along with monoclinic phase indicates the crystallographic rearrangement in ZrO_2_ due to the oxygen vacancies. The authors related the photocatalytic properties of ZrO_2_ with the trap levels present in it due to oxygen vacancies. It was observed that the photocatalytic response tracks the energy gap of the monoclinic phase which varies with the varying synthesis parameters.

Zhao et al. [17] used anodization method to synthesize ZrO_2_ nanotubes with a length of 25 μm, inner diameter of 80 nm, and wall thickness of 35 nm. The authors observed 97.6 decolorization percentage of methyl orange in 8 h at optimal pH value 2. Ismail et al. [18] synthesized 6-μm-thick anodic oxide film with nanotubular ZrO_2_ structure, and the authors tested the photocatalytic ability of the ZrO_2_ nanotubes. The authors reported 30% of methyl orange degradation under UV light in the presence of the cubic/tetragonal ZrO_2_ nanotubes after 120 min of reaction.

Jiang et al. [19] used zirconium foil to anodize in electrolyte containing 1 M (NH_4_)_2_SO_4_ and 0.25 wt.% NH_4_F to *in situ* construct the ZrO_2_ nanotubes on the surface. The authors reported that ZrO_2_ nanotubes showed excellent photocatalytic performance with methyl orange photodegradation rate of 94.4% after 240 min. They also claimed that photocatalysis performance was due to the hydroxyl group absorbing on the surface.

Shu et al. [20] synthesized tetragonal star-like ZrO_2_ nanostructures using hydrothermal synthesis method. The authors used ZrO_2_ nanostructures for the photodegradation of anionic dyes including methyl orange, in acidic, neutral, and weak basic aqueous solutions. They observed that the ZrO_2_ sample offered complete degradation of methyl orange within 60 min; however, authors have not studied the stability and reusability of the synthesized ZrO_2_ nanomaterial.

The objective of the present study is to synthesize nanocrystalline mesoporous monoclinic, tetragonal, and cubic ZrO_2_ samples with high surface area using fairly simple experimental procedures. In this work, nanosized pure monoclinic, tetragonal, and cubic ZrO_2_ samples were prepared and the physico-chemical properties of the samples were performed by different characterization techniques. The photocatalytic degradation of methyl orange over the three ZrO_2_ samples were studied and correlated to the phase structure, specific surface area, and electronic properties of the catalysts.

## Methods

### Materials

Zirconyl chloride, zirconium isopropoxide, sodium hydroxide solution, methyl orange, and hydrochloric acid were purchased from Aldrich, Dorset, England, UK. All chemicals used in this study were analytical grade and used directly without further purification. Deionzied water was used for the preparation of the methyl orange standard solution as well as the respective dilutions.

### Synthesis of pure monoclinic, tetragonal, and cubic ZrO_2_ samples

#### Monoclinic ZrO_2_

A near pure monoclinic nanocrystalline ZrO_2_ was synthesized by following the method reported by Guo et al. [21]. The zirconyl chloride was dissolved in deionized water so that the final concentration of zirconium was 38.7 g per liter (0.42 M). Of the zirconyl chloride solution, 15 ml was added to 300 mL deionized water in a glass beaker, and then concentrated aqueous ammonia was added rapidly to the solution with constant stirring until pH 4.5. The resultant precipitate was aged in the mother liquor for 24 h. After filtration, it was washed several times with dilute ammonia and hot deionized water (80°C) until chloride ions were no longer detectable in the washing water (AgNO_3_ test) and then dried at 100°C for 12 h. The synthesized sample was calcined at 500°C for 3 h in air with a ramp rate of 1°C min^−1^ and kept isothermally for 3 h and was annotated as *m*-ZrO_2_.

### Tetragonal ZrO_2_

Pure tetragonal ZrO_2_ was synthesized by the following reported procedure in the literature [22]. Zirconium oxychloride and ammonia solution (25% *w*/*w*) solutions were prepared using deionized water. First, 50 mL of 2.5 M ammonia solution was added to 50 mL of 0.1 M zirconium oxychloride solution drop by drop in a beaker and the mixture was stirred vigorously at room temperature for 4 h. The white zirconium hydroxide precipitates in time of addition of ammonia solution. The obtained precipitate was separated by centrifugation at 4,000 rpm, washed with water and ethanol for several times. Then, the precipitate was transferred into Teflon-lined autoclave, and the autoclave was kept at 100°C for 12 h. Finally, the white powder was calcined in furnace at 500°C for 3 h with a ramp rate of 1°C min^−1^ and kept at this temperature for 3 h and was annotated as *t*-ZrO_2_.

#### Cubic ZrO_2_

Cubic ZrO_2_ was synthesized by hydrothermal method reported my Tahir et al. [23]. In a Teflon vessel, 1 g of zirconium isopropoxide was dissolved in 6 mL of ethanol (99.8%) and then the Teflon vessel was kept in a desiccator containing a Petri dish filled with water at the bottom. The diffusion experiment was stopped after 12 h, followed by the addition of 25 mL of 10 M NaOH aqueous solution. Then, the reaction vessel was sealed into a stainless steel hydrothermal bomb, which was heated to 180°C for 18 h. After the autoclave was cooled down to room temperature, the products were filtered and repeatedly washed with 0.1 M HNO_3_, 1 N HCl, and deionized water. After drying under vacuum for 3 h, a white soft and fibrous powder was obtained. The obtained powder was calcined at 500°C for 3 h in air with a ramp rate of 1°C min^−1^ and kept isothermally for 3 h and was annotated as *c*-ZrO_2_.

### Material characterization

X-ray powder diffraction (XRD) studies were performed for all of the prepared solid samples using a Bruker diffractometer (Bruker D8 advance target; Bruker AXS, GmbH, Karlsruhe, Germany). The patterns were run with copper Kα_1_ and a monochromator (*λ* = 1.5405 Å) at 40 kV and 40 mA. The crystallite size of the ZrO_2_ was calculated using Scherrer's equation;

1 $$ D = B\lambda /{\beta}_{1/2} \cos \theta $$

where *D* is the average crystallite size of the phase under investigation, *B* is the Scherer constant (0.89), *λ* is the wavelength of the X-ray beam used (1.54056 A°), *β*_1/2_ is the full width at half maximum (FWHM) of the diffraction peak, and *θ* is the diffraction angle. The identification of different crystalline phases in the samples was performed by comparing the data with the Joint Committee for Powder Diffraction Standards (JCPDS) files.

The Raman spectra of the samples were measured with a Bruker Equinox 55 FT-IR spectrometer equipped with an FRA106/S FT Raman module and a liquid N_2_-cooled Ge detector using the 1,064-nm line of a Nd:YAG laser with an output laser power of 200 mW.

A Philips CM200FEG microscope (Philips, Amsterdam, The Netherlands), 200 kV, equipped with a field emission gun was used for HRTEM analysis. The coefficient of spherical aberration was C_*s*_ = 1.35 mm. The information limit was better than 0.18 nm. High-resolution images with a pixel size of 0.044 nm were taken with a CCD camera.

The textural properties of the prepared samples were determined from nitrogen adsorption/desorption isotherm measurements at −196°C using Autosorb automated gas sorption system (Quantachrome, Boynton Beach, FL, USA). Prior to measurement, each sample was degassed for 6 h at 150°C. The specific surface area, *S*_BET_, was calculated by applying the Brunauer-Emmett-Teller (BET) equation. The average pore radius was estimated from the relation 2*V*_p_/*S*_BET_, where *V*_p_ is the total pore volume (at P/P^0^ = 0.975). Pore size distribution over the mesopore range was generated by the Barrett-Joyner-Halenda (BJH) analysis of the desorption branches, and the values for the average pore size were calculated.

The X-ray photoelectron spectroscopy (XPS) measurements were carried out by using a SPECS GmbH X-ray photoelectron spectrometer (SPECS, Berlin, Germany). Prior to analysis, the samples were degassed under vacuum inside the load lock for 16 h. The binding energy of the adventitious carbon (C *1 s*) line at 284.6 eV was used for calibration, and the positions of other peaks were corrected according to the position of the C *1 s* signal. For the measurements of high-resolution spectra, the analyzer was set to the large area lens mode with energy steps of 25 m eV and in fixed analyzer transmission (FAT) mode with pass energies of 34 eV and dwell times of 100 ms. The photoelectron spectra of the four samples were recorded with the acceptance area and angle of 5 mm in diameter and up to ±5°, respectively. The base pressure during all measurements was 5 × 10^−9^ mbar. A standard dual anode excitation source with Mg K_α_ (1,253.6 eV) radiation was used at 13 kV and 100 W.

The UV-vis absorption spectra in transmittance mode were recorded on a Thermo Scientific (Evolution 600 UV-vis; Thermo Fisher Scientific, Waltham, MA, USA) instrument. The optical bandgap of the samples is measured by plotting *a* (*αhυ*)^2^ versus *hυ*. The extrapolation of the straight line in the graph to (*αhυ*)^2^ = 0 gives the value of the energy band gap.

### Photocatalytic degradation of methyl orange

Photocatalytic activity measurements were carried out in a homebuilt reactor. The reactor is a wooden box with dimensions of 100 cm height, 100 cm width, and 60 cm thickness, equipped with a 12-V transformer for an electric exhaust fan. Six 18 W UV lamps (60 cm × 2.5 cm) of approximately 350 to 400 nm (F20 T8 BLB) were used; the total power of the UV light at the surface of the test suspension measured with a Newport 918DUVOD3 detector (Newport Corporation, Irvine, CA, USA) and power meter was 13 W/m^2^. In a typical experiment, 100 mL of aqueous methyl orange solution (10 mg/L) was stirred (300 rpm) with 100 mg of the different photocatalysts. The resulting suspension was equilibrated by stirring for 1 h to stabilize the absorption of methyl orange dye over the surface of catalyst before exposing to the UV light. Samples were withdrawn at 10 min intervals, filtered through a 0.2-mm PTFE Millipore membrane filter (Millipore, Billerica, MA, USA) to remove suspended catalyst agglomerates, and finally analyzed using the UV-vis spectrometer (Thermo Fisher Scientific Evolution 160) in the range between 250 and 600 nm. The decolorization rate percentages of methyl orange were calculated by the following equation:

2 $$ \mathrm{Decolorization}\ \% = \left(1 - \frac{\mathrm{C}}{{\mathrm{C}}_{\mathrm{o}}}\right)\times 100 $$

where *C*_o_ is the concentration of methyl orange before illumination and *C* is the concentration after a certain irradiation time.

## Results and discussion

### X-ray powder diffraction

**Figure 1:**
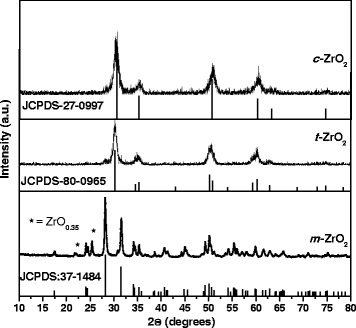
**XRD patterns of the different zirconia samples.**

The XRD patterns of synthesized *m*-ZrO_2_, *t*-ZrO_2_, and *c*-ZrO_2_ samples and corresponding JCPDS reference patterns are shown in Figure 1. XRD pattern of the *m-*ZrO_2_ sample showed intensive diffraction patterns at 2*θ* = 24.2°, 28.2°, 31.4°, and 34.3° which are corresponding to monoclinic ZrO_2_ crystal phase [JCPDS 37-1484]. It is observed that there is one major peak at 2*θ* = 25.4° and another small peak at 22°, which are not indexed for monoclinic ZrO_2_ phase. These peaks can be indexed to the oxygen-deficient zirconium oxide, ZrO_0.35_ phase [JCPDS; 17-0385, hexagonal, space group *P6322*]. To determine the purity of monoclinic phase of the *m*-ZrO_2_ sample, volume percent of monoclinic and oxygen-deficient zirconium oxide phase present in the *m*-ZrO_2_ sample was determined from the integrated intensities of the diffraction peaks (−111) (111) of *m*-ZrO_2_ at 2*θ* = 28.5° and 31.5°, respectively, and the diffraction line (101) of oxygen deficient ZrO_2_ at 2*θ* = 25.4°. We used the expressions (3) and (4) reported in the literature [24].

3 $$ \%{M}_{\mathrm{monoclinic}}=\varSigma\ {M}_{\mathrm{monoclinic}} \times 100\ /\ \mathrm{O}{\mathrm{D}}_{\mathrm{oxygen}\ \mathrm{deficient}}+\varSigma\ {M}_{\mathrm{monoclinic}} $$

4 $$ \%{\mathrm{OD}}_{\mathrm{oxygen}\ \mathrm{deficient}}=100 - \%{M}_{\mathrm{monoclinic}} $$

The percentages of monoclinic and oxygen deficient phases were found to be 97% and 3%, respectively, for the *m*-ZrO_2_ sample. There are no additional unindexed peaks in the *m*-ZrO_2_ sample.

XRD pattern of the *t*-ZrO_2_ sample showed peaks for pure tetragonal phase of ZrO_2_ [JCPDS 80-0965] at 2*θ* = 30.2°, 35.2°, 50.6°, and 60.2°. No additional peaks corresponding to any other phase was observed in XRD pattern of this sample. All of the diffraction peaks of the XRD pattern of the *c*-ZrO_2_ sample can be indexed to the standard pattern of the pure cubic phase of ZrO_2_. Peaks at 2*θ* = 30.3°, 35.14°, 50.48°, and 60.2° reveal the presence of (111), (200), (220), and (311) planes, respectively, of cubic ZrO_2_ according to JCPDS CAS number 27-0997. These observation indicates that the *m*-ZrO_2_ sample is near pure monoclinic; however, the *t*-ZrO_2_ and *c*-ZrO_2_ samples did not show presence of any additional phases or impurities indicating that these two phases are pure in composition. In addition, the intensities of diffraction peaks of the *m*-ZrO_2_ sample were much higher than those of the *t*-ZrO_2_ and *c*-ZrO_2_ samples indicating that the *m*-ZrO_2_ sample is highly crystalline than the *t*-ZrO_2_ and *c*-ZrO_2_ samples.

However, it is known that assignment of cubic and tetragonal structures, based solely on the X-ray diffraction analysis, can be misleading because the cubic and tetragonal structures (*a*_0_ = 0.5124 nm for cubic and *a*_0_ = 0.5094 nm and *c*_0_ = 0.5177 nm for tetragonal structures) are very similar [25]. The authors also reported that the tetragonal structure can be distinguished from the cubic structure by the presence of the characteristic splitting of the diffraction peaks, whereas the cubic phase exhibits only single peaks. A significant line broadening obscured any clear distinction between the tetragonal and cubic polymorphs of ZrO_2_ (Figure 1). However, this detection is possible by measuring with high step counting times the (112) Bragg reflection of the tetragonal structure, which is forbidden in the cubic symmetry [26]. As it can be observed in Figure 1, a shift of the peak positions to higher 2*θ* values occurred. This shift may indicate a decrease in the lattice parameters.

The crystallite size was calculated using the Scherrer's equation (1). The average crystallite sizes of the monoclinic phase, calculated from the (111) diffraction peak was found to be 34 nm. Similarly, the average crystallite sizes, calculated from the (111) diffraction peak of the tetragonal and cubic phases, were found to be 17 and 20 nm for the *t*-ZrO_2_ and *c*-ZrO_2_ samples, respectively.

### Raman spectroscopy

**Figure 2:**
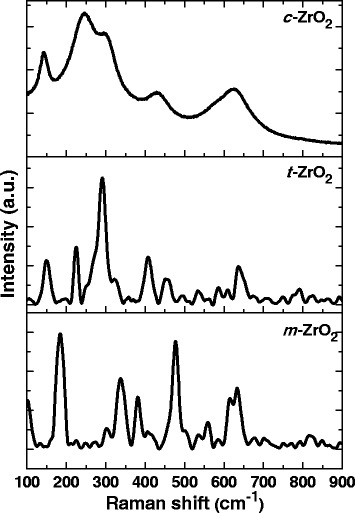
**Raman spectra of different zirconia samples.**

In order to confirm the crystalline structure of the samples, the Raman spectra of the samples were obtained and shown in Figure 2. From this figure, we can see that the *m*-ZrO_2_ sample showed several peaks centering at 183, 301, 335, 381, 476, 536, 559, 613, and 636 cm^−1^. The strong peaks are at 183, 335, and 476 cm^−1^. The exhibited bands are clearly indicating that the *m*-ZrO_2_ sample possessed dominant monoclinic phase of ZrO_2_ [27]. The *t*-ZrO_2_ sample showed peaks at 149, 224, 292, 324, 407, 456, and 636 cm^−1^, and the peak positions are in quite accordance with the reported values for tetragonal phase of ZrO_2_ [28].

The Raman spectrum for *c*-ZrO_2_ is characterized by a narrow band at 145 cm^−1^ and broad bands centered at 246, 301, 436, and 625 cm^−1^. Gazzoli et al. [29] reported that the Raman peak at 149 cm^−1^ is common for both of tetragonal and cubic phases, and cubic ZrO_2_ presents the strong band between 607 and 617 cm^−1^. The *c*-ZrO_2_ sample in this study clearly showed the broad peak centered at 625 cm^−1^, and this sample also shows poorly defined features related to the disordered oxygen sub-lattice whereas tetragonal ZrO_2_ exhibits several well-defined sharp bands because of the symmetry reduction [30]. In addition, the highly intense peaks at 292 and 636 cm^−1^ which are main characteristic bands of tetragonal ZrO_2_ cannot be found in the spectrum of the *c*-ZrO_2_ sample, which indicates the absence of tetragonal ZrO_2_ phase in this sample. Kontoyannis et al. [31] also reported that cubic ZrO_2_ shows amorphous-like Raman spectrum with one broad band at 530 to 670 cm^−1^. The features of Raman spectrum of *c*-ZrO_2_ as shown in Figure 2 is in accordance with the spectral results reported in the literature.

### Transmission electron microscopy

**Figure 3:**
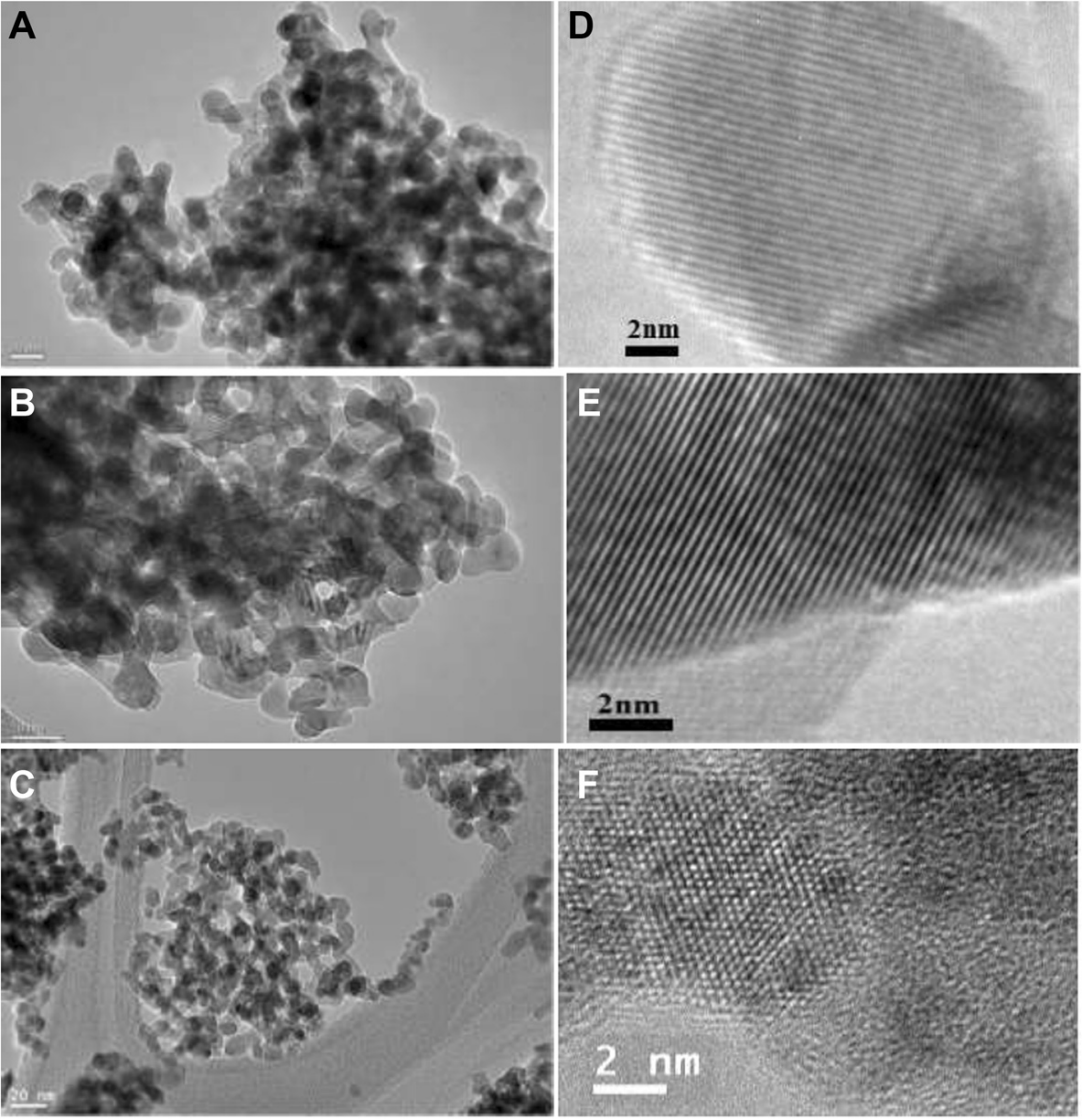
**TEM images of (A)** ***m*** **-ZrO**_**2**_ **(B)** ***t*** **-ZrO**_**2**_ **(C)** ***c*** **-ZrO**_**2**_ **and HRTEM images of (D)** ***m*** **-ZrO**_**2**_ **(E)** ***t*** **-ZrO**_**2**_ **(F)** ***c*** **-ZrO**_**2**_ **.**

The TEM images for the *m*-ZrO_2_, *t*-ZrO_2_, and *c*-ZrO_2_ samples are shown in Figure 3A,B,C, respectively. Tightly packed dumbbell-shaped particles can be observed in the low magnification TEM images of three samples. The average particle size for the *m*-ZrO_2_, *t*-ZrO_2_, and *c*-ZrO_2_ samples was found to be 24, 18, and 8 nm, respectively. There are conflicting reports in the literature regarding the phase structure of ZrO_2_ particles in smaller size (less than 10 nm). Some authors reported that cubic ZrO_2_ phase exists as fine nanoparticles [32], and few other researchers reported that tetragonal ZrO_2_ phase exists in smaller size than cubic phase [33]. However, in the present work, it is clear that in the *c*-ZrO_2_ sample, pure cubic ZrO_2_ phase possessed smaller particles size than the *t*-ZrO_2_ sample (pure tetragonal).

### High-resolution transmission electron microscopy

In order to authenticate the ZrO_2_ phase existed in the samples, high-resolution transmission electron microscopy (HRTEM) was carried out on particles of the three samples. Figure 3D represents the HRTEM image of the *m*-ZrO_2_ sample. The image clearly showed well-resolved lattice fringes. The distance between the fringes was calculated to be 0.297 nm which can be attributed to the interplanar spacing corresponding to (111) plane of monoclinic ZrO_2_ [34]. The HRTEM image of *t*-ZrO_2_ was shown in Figure 3E. This image also showed well-resolved equidistant lattice fringes. The distance between the parallel fringes was calculated to be 0.296 nm which can be attributed to the well-recognized lattice d-spacing of (111) plane of tetragonal ZrO_2_ [35]. A typical HRTEM image of particles of the *c*-ZrO_2_ sample is shown in Figure 3F. The image shows equidistant parallel fringes which depict single crystalline nature of the particle. The distance between the parallel fringes was calculated to be 0.291 nm which is the well-recognized lattice d-spacing of (111) plane of cubic ZrO_2_ [36].

### BET surface area

**Figure 4:**
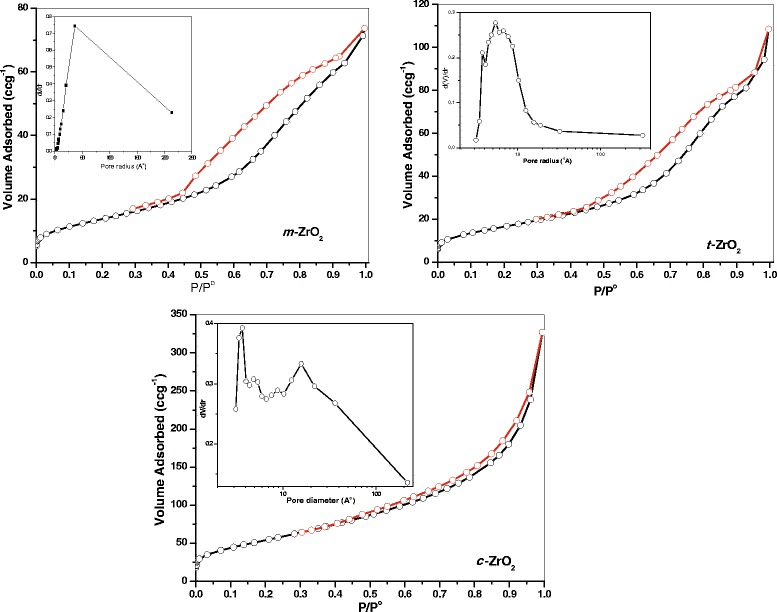
**Nitrogen adsorption-desorption isotherms and pore size distribution (inset) of the samples.**

A typical nitrogen adsorption-desorption isotherms of the samples are shown in Figure 4. The adsorption-desorption patterns of the three ZrO_2_ samples belong to the typical IUPAC IV-type with the H2-type hysteresis loop, which is a characteristic of particles with uniform size and mesoporous structure [37]. From the figure, it is clear that all the three samples showed type IV isotherms with hysteresis loop at P/P^o^ = 0.45 to 0.95. However, each sample exhibited a different type of hysteresis loop suggesting that pore size and shape were not same in these samples. The H2-type adsorption hysteresis can be explained as a consequence of the interconnectivity of pores. It was reported that in such systems, the distribution of pore sizes and pore shapes are not well defined or irregular. A sharp step on desorption isotherm is usually understood as a sign of interconnection of the pores. The shape of hysteresis loop of the *m*-ZrO_2_ sample suggesting that this sample possessed pores known as ‘ink-bottle’ type [38].

The pore size distribution patterns of the synthesized ZrO_2_ samples were shown in the inset of Figure 4. Pore size distribution of the *m*-ZrO_2_ sample reveals a broad and monomodal distribution of the pore dimension in the mesoporous region. In addition, the *c*-ZrO_2_ sample showed a narrower pore size distribution than *t*-ZrO_2_. The porosity of the *m*-ZrO_2_ and *t*-ZrO_2_ samples appears to be arising from non-crystalline intra-aggregate voids and spaces formed as a result of inter-particle contact [39]. The narrow and broad bimodal distribution of pores can be observed in the case of the *c*-ZrO_2_ sample. The porosity of this sample appears to be framework porosity which corresponds to the porosity within the uniform channels of ZrO_2_ structure.

The textural properties of the synthesized ZrO_2_ samples from the adsorption-desorption data was tabulated in Table 1. Specific surface area of the *m*-ZrO_2_ sample is 65 m^2^g^−1^, an average pore radius of 50 Å, and a total pore volume of 0.626 cm^3^g^−1^. The *t*-ZrO_2_ and *c*-ZrO_2_ samples possessed the surface area of 74 and 204 m^2^g^−1^; the drastic increase of the surface area of the *c*-ZrO_2_ sample could be due to very small size of particles (TEM results). However, the *c*-ZrO_2_ sample possessed pores with small radius (19 Å) than the *t*-ZrO_2_ (28.3 Å) and *m*-ZrO_2_ (50 Å) samples.

**Table 1:** **Textural properties of the catalysts from N**_**2**_ **adsorption measurements**

**Catalyst**	***S***_**BET**_ **(m** ^**2**^ **g** ^**−1**^ **)**	***V***_**p**_ **(cm** ^**3**^ **g** ^**−1**^ **)**	**Pore radius (Å)**
m-ZrO_2_	65	0.626	50.0
t-ZrO_2_	74	0.521	28.3
c-ZrO_2_	204	0.508	19.0

### X-ray photoelectron spectroscopy

**Figure 5:**
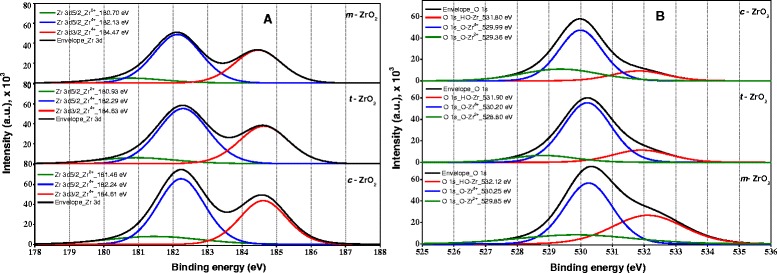
**Deconvoluted XPS spectra of samples (A) Zr (** ***3d*** **and** ***5d*** **) (B) O (** ***1 s*** **).**

It is known that XPS is a very sensitive tool in analyzing the chemical state of Zr cations in ZrO_2_ and its composites [40]. Figure 5A,B displays the XPS spectra of the Zr *3d* and O *1 s* core levels of the three samples, respectively. The peaks located at 181.3 and 183.8 eV are attributed to the spin-orbit splitting of the Zr *3d* components, Zr *3d*_*5/2*_ and Zr *3d*_*3/2*_. The binding energy of O *1 s* in ZrO_2_ is located at 530.1 eV.

Deconvolution of the spectra produces peaks attributed to the existence of two kinds of zirconium species, referred as Zr^2+^ species with low binding energy in the range 180.7 to 181.4 eV and Zr^4+^ species with higher binding energy in the range of 182.1 to 182.3 eV. It should be noted that the fraction of Zr^4+^ species for all samples is larger compared to that of species Zr^2+^. It is reported that the binding energy of Zr^4+^ species in pure ZrO_2_ is around 182.6 eV [41]; however, slightly lower values compared to that of stoichiometric ZrO_2_ were observed especially for the sample *m*-ZrO_2_ (182.1 eV), probably, due to some oxygen deficiency. The position shift toward the lower binding energy might be associated with the holes created by oxygen vacancies in the ZrO_2_ lattice [42].

Kawasaki [43] reported that Zr *3d* components, Zr *3d*_*5/2*_ and Zr *3d*_*3/2*_ for cubic ZrO_2_ can be observed at 182.0 and 184.4 eV, respectively. The same components for tetragonal ZrO_2_ and monoclinic ZrO_2_ samples appeared at 182.7 and 184.7 eV [44] and 182.2 eV and 184.6 eV [45], respectively. The binding energy values of Zr *3d* components observed in this study are in accordance with values reported in the literature.

The O *1 s* broad peaks can be deconvulated into three peaks at the corresponding position using XPS Casa Software, whose relative contents are shown in Table 2. Navio et al. [9] observed two types of oxygen species in the ZrO_2_ sample, oxygen species of ZrO_2_ and oxygen species of Zr-OH, whose binding energy is in the range of 529.8 to 530.3 and 530.9 to 532.2 eV, respectively. It was also reported that the oxygen species with binding energy 531.0 eV are attributed to Zr-OH groups [46]. All the three samples showed XPS peaks corresponding to Zr-OH, Zr^4+^-O, and Zr^2+^-O species in different proportions [43]. The *m*-ZrO_2_ sample showed highest 12.3 mass percentage Zr-OH groups bounded to Zr atom, while *t*-ZrO_2_ and *c*-ZrO_2_ have 8% and 6.1%, respectively. These features of the XPS spectra indicate that the *c*-ZrO_2_ and *t*-ZrO_2_ samples were regular surfaces and with no apparent defect relative to the Zr^4+^ species; this is rather significant. In fact, the *m*-ZrO_2_ sample showed surface defects with more surface hydroxyl groups.

**Table 2:** **Surface composition of catalysts from XPS measurements**

**Catalyst**	**Zr (mass%)**		**O (mass%)**		
	**Zr** ^**4+**^	**Zr** ^**2+**^	**O-Zr** ^**4+**^	**O-Zr** ^**2+**^	**Zr-OH**
m-ZrO_2_	52.7	13.0	16.3	5.7	12.3
t-ZrO_2_	53.9	14.3	17.3	6.5	8.0
c-ZrO_2_	54.1	15.1	17.6	7.1	6.1

### Diffuse-reflectance UV-vis

**Figure 6:**
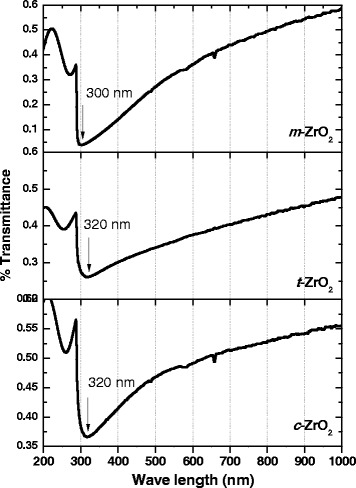
**UV-vis absorption spectra of the samples.**

Figure 6 represents the UV-vis absorption spectra of the three ZrO_2_ samples. It is known that all ZrO_2_ polymorphs are very similar in vibrational structure, and a minor variation in their band frequencies or intensities infers small differences in the Zr^4+^ distribution in Zr-O sites and the oxygen vacancies and other structural defects [47]. Herrera et al. [48] reported that UV-vis spectra of monoclinic Fe-doped ZrO_2_ display two bands at around 245 and 320 nm, which are associated with charge transfer transitions.

Li et al. [49] indicated that the ZrO_2_ sample with pure monoclinic ZrO_2_ nanoparticles showed a pronounced absorption peak at 270 nm, and tetragonal and cubic ZrO_2_ nanoparticles show an absorption peak at 314 nm. The absorptions in the range 250 to 350 nm were assigned to O_2_ → Zr^4+^ charge transfer transitions with Zr in low coordination states (possibly six) either isolated or present in small Zr*x*O*y* clusters [10].

Band gap of all the ZrO_2_ samples was determined by establishing the relation between *hυ* and (*αhυ*)^2^. The obtained data indicated that the band gap energy for *m*-ZrO_2_ (3.25 eV) is lower compared with *t*-ZrO_2_ (3.58 eV) and *c*-ZrO_2_ (4.33 eV). Crystal structure plays an important role in the electronic structure of ZrO_2_. This effect is most significant in the *d*-electron-derived conduction bands (CBs). It is reported that the reduction of the CB gap between the Zr *4d* (*x*2*−y*2, *z*2) and the Zr *4d* (*xy*, *yz*, *zx*) CBs is present in cubic ZrO_2_ and disappears in tetragonal ZrO_2_, and also, substantial volume expansion was observed in the case of monoclinic ZrO_2_ due to the hybridization of Zr *4d* CBs into a new single Zr *4d* CB [50].

It was also reported that the pure tetragonal and monoclinic ZrO_2_ nanoparticles showed energy band gaps of 4.0 and 3.5 eV, respectively [47]. These values are very similar to the values reported in the literature reports. Emeline et al. [51] determined an energy band gap of 5.0 eV for monoclinic ZrO_2_ thin films calcined at 550°C, and Chang and Doong [52] determined an energy gap of 5.7 eV for the same sample at the same temperature. However, Navio et al. [9] reported an energy band gap of 3.7 eV for monoclinic ZrO_2_ powders prepared by sol-gel method. These authors claim that the decrease in the band gap energy could be attributed to a highly disordered structure, as a result of the conditions used in the preparation technique. As a consequence of structural defects, some energy levels are introduced into the semiconductor band gap that allow transitions of lower energy and therefore lead to a decrease of the band gap energy.

### Photocatalytic degradation of methyl orange

**Figure 7:**
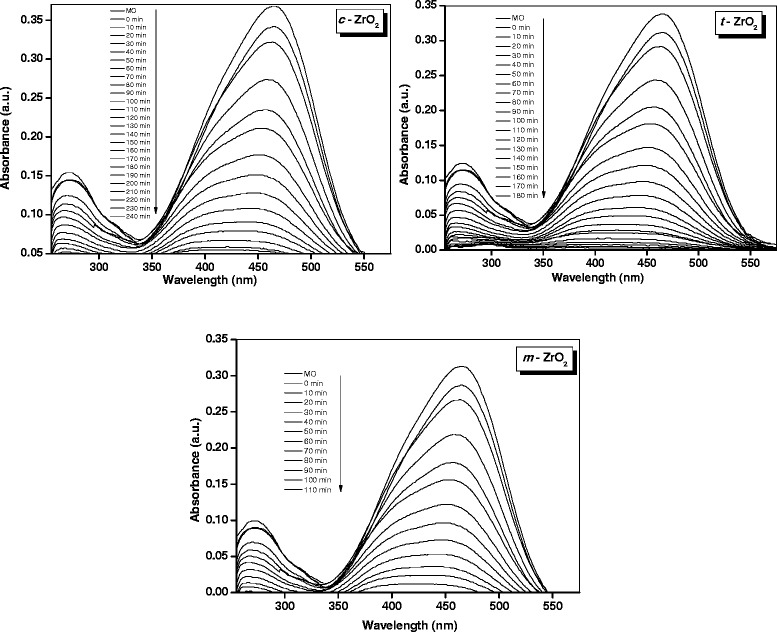
**UV-visible absorption changes of methyl orange aqueous solution at 25°C in the presence of the ZrO**_**2**_ **samples.**

The photocatalytic activity of the three ZrO_2_ samples was determined by monitoring the degradation of the methyl orange dye. A blank experiment was carried out to confirm that the photo-degradation reaction did not proceed without the presence of either catalyst or the UV radiation. Figure 7 shows the change in the UV-vis absorbance spectra of methyl orange solution (10 ppm) with different irradiation intervals over the ZrO_2_ samples.

As mentioned in the experimental section, the catalyst was equilibrated with the methyl orange solution to check for adsorption of the dye on the solid photocatalyst. The spectra depicted in Figure 7 were recorded after the equilibration of the photocatalyst. The UV-vis absorption spectra of methyl orange have a strong characteristic peak at 465 nm and a weak absorption peak at 274 nm.

**Figure 8:**
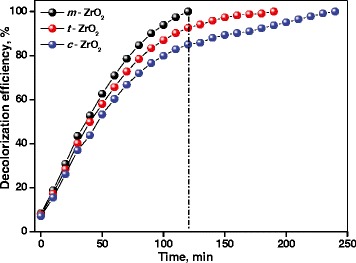
**Decolorization efficiency of methyl orange aqueous solution at 25°C in presence of ZrO**_**2**_ **samples.**

These absorption peaks become weak and disappear along with the extension of reaction time. The UV-vis results indicate that methyl orange was degraded during the reaction. The decrease in the absorbance of the solution was due to the destruction of the homo- and hetero-polyaromatic rings present in the dye molecules. The *m*-ZrO_2_ sample was found to be the most effective catalyst in comparison with the *t*-ZrO_2_ and *c*-ZrO_2_ samples under identical optimized conditions (Figure 7). Complete degradation of adsorbed dye molecules was observed within 110 min for the *m*-ZrO_2_ sample, whereas 180 and 240 min were required for the complete the degradation of adsorbed dye molecules for the *t*-ZrO_2_ and *c*-ZrO_2_ samples under the similar conditions, respectively. The decolorization efficiency of the ZrO_2_ samples was calculated using Equation 2. Figure 8 showed degradation efficiency of methyl orange aqueous solution at 25°C in the presence of the three ZrO_2_ samples. The *m*-ZrO_2_ photocatalyst showed 99% degradation of methyl orange in 110 min of reaction; however, the *t*-ZrO_2_ and *c*-ZrO_2_ catalysts showed 90% and 80% degradation in the same reaction time, respectively.

It was reported that the structures of nanoscale favor the movement or transfer of electrons and holes generated inside the crystals to the surface [53], which also enhances the photocatalytic activity. The photocatalytic activity of ZrO_2_ appears to be strongly dependent on the surface composition. Bachiller-Baeza et al. [54] reported that Lewis acid sites were more abundant on monoclinic ZrO_2_ than on tetragonal ZrO_2_, and the former brought about stronger surface adsorption sites concerning CO_2_ adsorption than the latter. Ma et al. [55] determined and compared the surface properties of ZrO_2_ polymorphs. It was found that ZrO_2_ polymorphs exhibited different surface hydroxyl and acid-base properties. These differences had great influence on the behavior of CO adsorption and reaction. They also showed that monoclinic ZrO_2_ possessed better adsorption properties than the other ZrO_2_ structures.

The adsorption capacity of methyl orange per gram of each catalyst was determined under identical conditions. It was observed that *m*-ZrO_2_ (1.95 mg g^−1^) possessed better adsorption capacity than the *t*-ZrO_2_ (1.05 mg g^−1^) and *c*-ZrO_2_ (0.65 mg g^−1^) samples. The photocatalytic activities observed in this study show similar trend that the *m*-ZrO_2_ sample showed high photocatalytic activity even though it possessed less surface area. ZrO_2_ nanomaterials have been investigated previously in the photocatalytic degradation of methyl orange; the time period required for the degradation of methyl orange tends to be 120 min or greater. Here, the synthesized pure monoclinic ZrO_2_ nanoparticles offered 99% of efficiency in 110 min of reaction time. The observed photocatalytic degradation activity is substantially higher than the activity reported for the ZrO_2_ samples in the literature.

It is known that usually high surface area of photocatalyst enhances dye adsorption and subsequent photocatalytic activity. However, it was also reported that the amount of dye adsorption on a catalyst also depends on the adsorption coefficient. Thus, a high adsorption coefficient on a low surface area material could lead to the same amount of adsorbed material per gram of catalyst as low adsorption coefficient on a high surface area material. This could be the reason why the rate (per gram basis) of methyl orange degradation is high for *m*-ZrO_2_ (low surface area) and *c*-ZrO_2_ (high surface area).

The *m*-ZrO_2_ sample possesses the smallest surface area (65 m^2^g^−1^) and largest pore size, while the *c*-ZrO_2_ has the largest surface area of 204 m^2^g^−1^ and smallest pore size. It is interesting that *m*-ZrO_2_ exhibited higher photcatalytic activity than *c*-ZrO_2_. Guo et al. [56] studied the influence of the pore structure of TiO_2_ on its photocatalytic performance. They observed that the photocatalytic activity of nanometer TiO_2_ is less than that of mesoporous TiO_2_.

Shao et al. [57] synthesized ZrO_2_-TiO_2_ composites which possessed different surface areas and textural properties. Composite samples showed surface area in the range of 270 to 80 m^2^g^−1^; however, the sample which has low surface area showed high photocatalytic activity due to it optimum Ti-Zr composition. It is clear from the literature reports that it is possible for catalyst, which possessed low surface area, to offer better photocatalytic activity than the catalyst which possessed high surface area. This is due to the fact that photocatalytic activity can be influenced by several other factors such as crytallinity, composition, particle size distribution, porosity, band gap, and surface hydroxyl density [58].

The process of photocatalytic degradation of methyl orange over ZrO_2_ catalysts can be described as follows. The first step involves adsorption of the dye onto the surface of ZrO_2_ nanostructure sample. Exposure of dye adsorbed ZrO_2_ nanostructures with UV light leads to generation of electron-hole (e^−^-h^+^) pairs in ZrO_2_ as indicated in Equation 5. The photogenerated electrons in the conduction band of ZrO_2_ interact with the oxygen molecules adsorbed on ZrO_2_ to form superoxide anion radicals (*O_2_^−^) (Equation 6). The holes generated in the valence band of ZrO_2_ react with surface hydroxyl groups to produce highly reactive hydroxyl radicals (*OH) (Equation 7). These photogenerated holes can lead to dissociation of water molecules in the aqueous solution, producing radicals (Equation 8). The highly reactive hydroxyl radicals (*OH) and superoxide radicals (*O_2_^−^) can react with methyl orange dye adsorbed on ZrO_2_ nanostructures and lead to its degradation as represented in Equations 9 and 10.

5 $$ \mathrm{Z}\mathrm{r}{\mathrm{O}}_2 + h\upsilon \to {\mathrm{e}}^{-}\left(\mathrm{C}\mathrm{B}\right) + {\mathrm{h}}^{+}\left(\mathrm{V}\mathrm{B}\right) $$

6 $$ {\mathrm{O}}_2+{\mathrm{e}}^{-}\to *{\mathrm{O}}_{2^{-}} $$

7 $$ {\mathrm{h}}^{+}+\mathrm{O}{\mathrm{H}}^{-}\to *OH $$

8 $$ {\mathrm{h}}^{+} + {\mathrm{H}}_2\mathrm{O}\to {\mathrm{H}}^{+}+*\mathrm{O}{\mathrm{H}}^{-} $$

9 $$ *\mathrm{O}{\mathrm{H}}^{-} + \mathrm{Methyl}\ \mathrm{orange}\kern0.5em \to \kern0.5em \mathrm{Degradation}\ \mathrm{products} $$

10 $$ *{\mathrm{O}}_2^{-} + \mathrm{Methyl}\ \mathrm{orange}\kern0.5em \to \kern0.5em \mathrm{Degradation}\ \mathrm{products} $$

**Figure 9:**
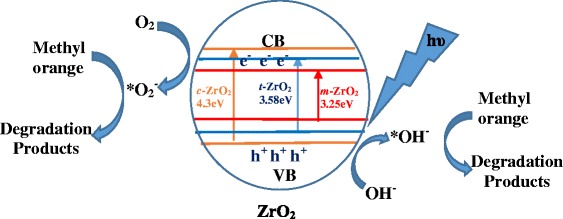
**Schematic representation of the processes leading to photocatalytic degradation.**

The processes leading to photocatalytic degradation of methyl orange and the mechanism over the mesoporous ZrO_2_ nanostructures were represented in Figure 9. It was reported that the enhanced photocatalytic activity of mesoporous structure to crystalline ZrO_2_ nanomaterial was due to the light harvesting capability, prolong the life of the photoinduced electron-hole pairs, and facilitate the reactant accessibility to surface active sites [11]. In the photocatalytic degradation process, the increase in photocatalytic activity is associated with efficient separation of photogenerated electrons and holes. If a surface defect state is able to trap electrons or holes, recombination can be restricted. The presence of oxygen-deficient ZrO_0.35_ impurity in the *m*-ZrO_2_ sample could also be responsible for the oxygen vacancies and they are acting as electron accepters to trap electrons and interstitial oxygen act as shallow trappers for holes, both of which prevent the recombination of photogenerated electrons and holes, thereby increasing the efficiency in the *m*-ZrO_2_ sample. In addition, the *m-*ZrO_2_ sample clearly possess the large pores, which can effectively facilitate both higher reactant accessibility to the surface active sites and more efficient multiple light scattering inside the pore channels [59].

**Figure 10:**
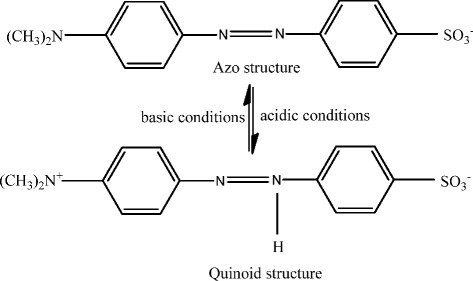
**Structural forms of methyl orange under acidic and basic conditions.**

It is also known that the photocatalytic redox reaction mainly takes place on the surface of the photocatalysts and so the surface properties significantly influence the efficiency of catalyst [60]. Additionally, the surface hydroxyl groups of ZrO_2_ are acidic to a certain degree, and the proportion of transformed azo structure increases into quinoid structure under acidic conditions (Figure 10). It is also reported that quinoid structure is more likely to be degraded than azo structure [61]. From the XRD patterns, it is clear that the *m*-ZrO_2_ sample has the highest crystallinity (sharpest peaks, largest crystals). This is also reflected in the Raman spectra. A higher crystallinity is usually believed to be beneficial for photocatalysis because of the amount of defect sites in the structure (which usually act as recombination centers). In comparison, the *m*-ZrO_2_ sample synthesized in this work possessed relatively high pore volume, pore size, and high density of hydroxyl groups.

We also tested the reusability of the *m*-ZrO_2_ catalyst for subsequent cycles of methyl orange degradation under optimized reaction conditions. Many of the reported photocatalysts have not been used for further degradation studies due to the fact that they undergo photocorrosion, by the direct illumination with light, and hence their photostability is diminished for further runs. For the reusability study, we collected the white-colored catalyst remained after the reaction, washed, dried at 100°C for 30 min, and used it for further reactions. The catalyst was found to be active for 5 cycles without any major deactivation, and more than 95% degradation was achieved in all experiments within 110 min using the *m*-ZrO_2_ catalyst. The reusability of the *m*-ZrO_2_ nanoparticles was ascribed to the low photocorrosive effect and high catalytic stability of the synthesized *m*-ZrO_2_ sample.

## Conclusions

Nanosize crystalline porous ZrO_2_ nanoparticles with pure monoclinic, tetragonal, and cubic phases were synthesized by different preparation methods. The photocatalytic performance of the three ZrO_2_ samples for the degradation of methyl orange was evaluated. Under the optimized reaction conditions, the *m*-ZrO_2_ sample comparatively showed a higher methyl orange degradation activity than the *t*-ZrO_2_ and *c*-ZrO_2_ samples. The pronounced photocatalytic activity for *m*-ZrO_2_ catalyst was mainly attributed to combining effects of factors including the presence of small amount of oxygen-deficient zirconium oxide phase, high crystallinity, broad pore size distribution, and high density of surface hydroxyl groups.
